# Nutraceutical Potential of Grape (*Vitis vinifera* L.) Seed Oil in Oxidative Stress, Inflammation, Obesity and Metabolic Alterations

**DOI:** 10.3390/molecules28237811

**Published:** 2023-11-28

**Authors:** Carolina Di Pietro Fernandes, Lidiani Figueiredo Santana, Jair Rosa dos Santos, Dayane Stéphanie Fernandes, Priscila Aiko Hiane, Arnildo Pott, Karine de Cássia Freitas, Danielle Bogo, Valter Aragão do Nascimento, Wander Fernando de Oliveira Filiú, Marcel Arakaki Asato, Rita de Cássia Avellaneda Guimarães

**Affiliations:** 1 Graduate Program in Health and Development in the Central-West Region of Brazil, Federal University of Mato Grosso do Sul, Campo Grande 79070-900, Brazil; nutricaroldipietro@gmail.com (C.D.P.F.); daystephaniefernandes@gmail.com (D.S.F.); priscila.hiane@ufms.br (P.A.H.); karine.freitas@ufms.br (K.d.C.F.); danielle.bogo@ufms.br (D.B.); aragao60@hotmail.com (V.A.d.N.); rita.guimaraes@ufms.br (R.d.C.A.G.); 2 State University of Mato Grosso do Sul (UEMS), Dourados 79804-970, Brazil; jair@uems.br; 3 Laboratory of Botany, Institute of Biosciences, Federal University of Mato Grosso do Sul, Campo Grande 79070-900, Brazil; arnildo.pott@gmail.com; 4 Pharmaceutical Science, Food and Nutrition Faculty, Federal University of Mato Grosso do Sul, Campo Grande 79070-900, Brazil; wander.filiu@gmail.com; 5 Medical School, Federal University of Mato Grosso do Sul, Campo Grande 79070-900, Brazil; marcel_arakakiasato@hotmail.com

**Keywords:** plant oil, linoleic acid, phenolics, resveratrol, therapeutic potential, oxidative stress, inflammation, obesity

## Abstract

*Vitis vinifera* L. (grapevine) is a perennial plant of the Vitaceae family that is widely used to produce grapes and wines. Grape seed oil is rich in fatty acids such as linoleic acid (65–75%), vitamin E (50 mg), and phytosterols in addition to phenolic compounds, such as catechins (414 mg), epicatechins (130.4 mg), and gallic acid (77 µg), shows promise as a nutritional compound and is outstanding as a therapeutic substance with active properties for health, detected mainly by in vitro studies, as well as some in vivo studies. The benefits of consuming this oil include modulating the expression of antioxidant enzymes, anti-atherosclerotic and anti-inflammatory effects, and protection against oxidative cell damage and some types of cancer. However, experimental findings confirm that therapeutic functions remain scarce; thus, more studies are needed to determine the mechanisms of action involved in the indicated therapeutic qualities.

## 1. Introduction

The Vitaceae family has about 15 genera and 800 species that are present in temperate regions such as Europe, most of North America, western Asia, southern South America, and the extreme north and south of Africa [1]. The species *Vitis vinifera* L. is produced by the vine (*Vitis* sp.) and is cultivated to produce wine and the fresh consumption of fruit in Europe. This vine of the Vitaceae family has the grape as fruit, which has been cultivated for thousands of years by different civilizations [2].

The commercialization and cultivation of grapes have been going on for more than two millennia in Western Europe and more than four millennia in the Eastern Mediterranean, with genetic alterations resulting from spontaneous reproduction in addition to the definition of methods for the genetic preservation of those most accepted and preferred by the local population. Thus, the grape varieties produced in Western Europe are currently the basis of the global wine industry, the main planting varieties being Cabernet Sauvignon, Merlot, Grenache, Sauvignon Blanc, Tempranillo, Chardonnay, Syrah, Pinot Noir, Airen, and Trebbiano Toscano [3].

Grape production has many specifications regarding the existing subspecies of this fruit, such as the presence or absence of seeds, different colors (reddish, black, and white), as well as fruit size and shape [4]. Therefore, there are beneficial nutritional properties present in parts of the plant, such as the root, stem, leaf, seed, and pulp, that can be used by the food industry to contribute to the health–disease process in different ways [5,6,7].

Various nutritional constituents have also been found in grapes (*V. vinifera* L.). The nutritional content of grapes includes proteins, lipids, carbohydrates, minerals, and vitamins. Each part of the vines or any other grape-based product contains different nutrients. Grape seeds (*Vitis vinifera* L.) have in their nutritional composition lipids (10.45–16.73 g), proteins (8.7–9.8 g), carbohydrates (18.2–19.8 g), and fiber (40.2–43.7 g) per 100 g [1,8,9].

The composition of *V. vinifera* seeds comprises 90% unsaturated fatty acids, with the major ones being linoleic fatty acid (65–75%) and oleic fatty acid (20–40%), and 10% saturated fatty acids [8,9], flavonoids (59.7 g/100 g), catechins (414 mg/100 g), procyanidins (2.5 g/100 g), phenolic compounds (73 μg/100 g) and tannins (14 mg CE/100 g); thus, they have antioxidant activity (1390.6 mMT/100 g) [10,11,12,13,14].

Grape seeds have shown antioxidant, antifungal, anti-inflammatory, and anti-obesity effects, as well as anticholinergic cicatrizing action [15]. Grape seed oil has the capacity to preserve important compounds to prevent oxidative stress, such as ascorbic acid, β-carotene, phytosterols, and tocopherols due to the presence of procyanidins in the plant’s stems and leaves; procyanidins in the seeds also indicate the antioxidant activity of the oil, according to Goufo, Singh and Cortez (2020) [16].

Grape seeds have health benefits, as scientific studies have demonstrated different biological and medicinal characteristics [15,16]. However, few studies have reported on their therapeutic potential for metabolic dysfunctions through experimental models of obesity, oxidative stress, or inflammation. Thus, our review investigated the nutritional value and bioactive compounds of *V. vinifera* seeds, in addition to ethnobotanical knowledge and possible metabolic, antioxidant, and anti-inflammatory applications.

## 2. Contextual Relationship of *Vitis vinifera* L.

Growing predominantly in regions with temperate and subtropical climates in the Northern Hemisphere [17], the European vine *V. vinifera* is considered the most relevant species and the most planted worldwide to meet the market demand for fine wines, sparkling wines and fresh fruit [18]. The vine (from the Latin *viere*, to fix) is a shrubby climber whose growth is controlled by pruning for grape quantity and quality [19]. 

Generally, grapes have a bunch of axes, juice, pulp, peel, and seeds [7]. The seeds represent about 10–12% of the solid residue left by the vinification process [20], and studies have shown that processing protocols can affect the content of seeds [21,22]. However, phenols and other components of grape seeds can still be used as nutraceuticals or can prevent diseases, which promotes the full utilization of grapes by reusing the non-edible parts [23].

### Nutritional Phytochemical Composition of Vitis vinifera L. Seed Oil

Grape (*V. vinifera*) seeds contain approximately 17% lipids (10.45–16.73 g/100 g), 10% proteins (8.7–9.8 g/100 g), 20% carbohydrates (18.2–19.8 g/100 g) and between 40 and 44% food fiber (40.2–43.7 g/100 g) (Table 1) [8,9].

Grape pomace is a solid organic by-product consisting of grape skin, stem fractions, pulp, and seed, which are discarded in the manufacture of wines or juices and can either be fermented or not [25]. The pomace powder presents amounts of moisture (8.9 ± 0.08), ash (24.97 ± 2.4), carbohydrates (6.13 ± 0.01), total fiber (2.16 ± 0.01), fat (7.69 ± 0.02), and crude protein (50.33 ± 2.1) [26]. Only the grape seeds are also food residues and have in their composition in oil a mean 90% of unsaturated fatty acids (Table 2), mainly linoleic (65–75%) and oleic (20–40%) fatty acids, and 10% saturated fatty acids [8,9,14]. 

*Vitis vinifera* seed oil presents a rich variety of macro- and microelements, including potassium (4347.8–9492.6 mg/1000 g), phosphorus (2277.6–3232.4 mg/1000 g) and calcium (1249.1–2073.9 mg/1000 g) (Table 3) [24].

Regarding vitamins, Goufo et al. determined that 50 mg of vitamin E exists per 100 g of grape seed and noted that homologs α and β were the most abundant, representing 86–244 and 38–48 mg/1000 g, respectively, along with γ-tocopherols (17–29 mg/1000 g); regarding to-cotrienols, isomer γ (499–1575 mg/1000 g) was the most abundant (Table 4) [16]. Among the tocopherols in grape seeds, ÿ-tocopherol (47% tocochromanols) and ÿ-tocotrienol (155 µg/g oleoresin) were the most abundant [31]. A study by Harbeoui et al. [32] analyzed the unsaponifiable fraction of seed oil in three varieties (Merlot, Carign and Syrah), showing the presence of two triterpenic compounds (β-mirin, lanosterol), six phytosterols (campesterol, ∆ 7-avenasterol, stigmasterol, β-sitosterol, β-sitostanol, cholesterol) and three tocopherols (α, β and γ). Dordevski et al. [14], exploring the functional properties of seed oil in Tamjanika, a subspecies of V. vinifera, found high quantities of tocotrienols (85.04 mg/100 g), predominating over tocopherols (8.37 mg/100 g), and also reported a carotenoid content of 0.27 mg/100 g, with lutein being the primary pigment.

Keskin et al. [33] reported a vitamin C level of 128.30 mg per 1000 g in pomace; however, in other studies, the content varied from 46.0 to 179.2 mg per 1000 g (Table 4) [34]. β-carotene content was reported to be between 33.9 and 59.8 ppm in the oil of grape seeds [35].

Evaluations of the phytochemical composition of grape seeds detected proanthocyanidins, i.e., oligomers and polymers of flavan-3-ol monomers, which presented only in the form of the procyanidins catechin, epicatechin and epicatechin gallate, and were linked mainly through C4–C6 or C4–C8 (type B) by differences in their constitutive units, the position of their connection and shape, and the polymerization degree [32]. Simpler and more common components of grape seeds include dimeric and trimeric procyanidins [36].

Bocsan et al. [13] identified a total polyphenol content in *V. vinifera* seed oil of 0.75 mg gallic acid equivalent (GAE)/100 g. By quantifying and qualifying the phenolic compounds extracted from the oil, they observed more catechins, epicatechins, gallic acid, quercetin, rutin, caffeic acid, procyanidins, and phenolic acids (Table 4) [37,38,39].

Mota et al. [16] analyzed the chemical profile of the seed oil of four cultivars of table grapes (*V. vinifera)*, and among the phenolic compounds identified 7.7 µg/g of gallic acid at a wavelength of 250 nm, and in the cultivars Cardinal (29.7 µg/g) and Muscat Hamburg (18.3 µg/g). 

## 3. Medicinal Properties of *Vitis vinifera* L. Seed Oil

**Figure 1:**
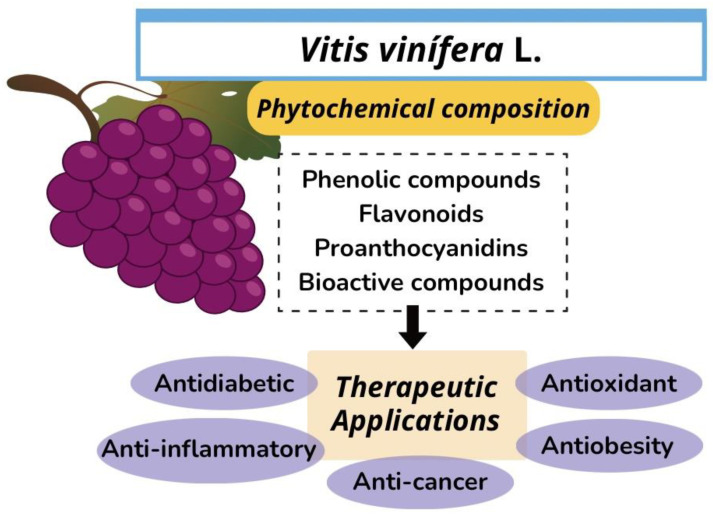
Phytochemical properties and therapeutic applications of grape (*Vitis vinífera* L.) seed oil.

The medicinal properties attributed to grapes include the protective effects of antioxidant, antifungal, anti-inflammatory, and anti-obesity compounds and anticholinergic and cicatrizing actions [27]. Grape seed oil can function in the preservation of essential compounds to prevent oxidative stress. Goufo et al. [16] reported the preservation of micronutrients such as ascorbic acid, tocopherol, and β- carotene due to the presence of procyanidins in the plant’s stems and leaves; thus, the presence of procyanidin in seeds can also indicate the antioxidant activity of the oil (Figure 1). 

Harbeoui et al. [32] used the unsaponifiable fraction to investigate cytotoxicity and cell viability. They observed a reduction from 10 to 15% of metabolically active cells in the cell culture in a time and dose-dependent manner and showed significant results in the DPPH trial after 24 h of incubation, demonstrating that the compounds present (ascorbic acid, tocopherol and β-carotene) in this fraction contribute to the potent and antiradical action of grape seed oil in addition to helping to protect against oxidative harm by modulating the production of nitric oxide (NO) and showing antioxidant activity.

Concerning the therapeutic properties, reports have indicated the use of seeds as well as leaves, stems, and fruits; however, the hypolipidemic action of grape seed oil stands out, with the dose for human consumption of one spoonful a day replacing fats and other oils [14]. 

Anti-inflammatory [40] and antidiabetic [41] activities have been reported since phenolic compounds (Figure 1), mainly catechins, play a relevant role in the inhibition and oxidation of cholesterol and low-density lipoprotein (LDL-c) and plate aggregation, increasing the concentration of antioxidant enzymes such as dismutases; in addition, the polyphenols of grape seeds can also inhibit inflammation and allergic reactions, through the action in some enzymes that catalyze histamine liberation [42].

Procyanidins present in grape seeds at concentrations of 2, 4, and 8 mL/kg contribute to the treatment of circulatory disturbances and promote anti-ulcerative activity by eliminating free radicals in the gut mucosa, inhibiting lesions [43]. 

Changes in fatty acid metabolism can promote the excessive lipidic peroxidation of LDL-c and the consequent development of cardiovascular disease. These oxidation products are also involved in the formation of thromboxane, which leads to increased plate aggregation, affecting thrombosis. By contrast, polyphenols reduce cardiovascular risk by promoting LDL-c lipidic oxidation [44].

Garavaglia et al. [45] reported that the anti-inflammatory, antioxidant, cardioprotective, and anti-cancer properties of grape seed oil may be due to linoleic acid, tocopherol, carotenoids, and phytosterols along with polyphenolic compounds such as proanthocyanidins, resveratrol, and quercetin. Furthermore, these polyphenolic compounds protect against the oxidation of ascorbic acid, selenium, and carotenoids and act to inhibit the enzymatic systems producing free radicals associated with inflammatory reactions, primarily cumaric, caffeic, ferulic, chlorogenic, neo-chlorogenic, p-hydroxybenzoic, vanillic and gallic acids [46].

Nevertheless, despite the solid background in the literature regarding the benefits of consuming table grapes, it is challenging to attribute these advantages to a particular compound or group of compounds since each subspecies contains various levels of phenolic compounds [47]. 

Seeds of the variety Globo Vermelho, for example, are considered to be a source of anthocyanins, flavones, flavonols and stilbenes (resveratrol), in addition to having microbicide activity against six bacterial species (*Staphylococcus aureus*, *Micrococcus flavus*, *Bacillus cereus*, *Pseudomonas aeruginosa*, *Escherichia coli* and *Enterobacter cloacae*) and dermatomycetes, as well as a potential antifungal role [48].

## 4. Antioxidant and Anti-Inflammatory Effects of Grape Seed Oil

Free radicals are atoms or molecules that contain one or more mismatched electrons in their last layer and are constantly produced in organisms. They provide typical reactivity and can occur, such as in various situations of mitochondrial metabolism, in the uric acid pathway (the enzyme xanthine oxidase), peroxisome activity, inflammation, phagocyte activity, the ischemia process, and physical exercise [49,50]. Some external factors, such as tobacco smoke, pollution, radiation, medicaments, pesticides, and industrial solvents, influence free radical levels and, when in excess, lead to oxidative stress, harming the organism [51].

**Figure 2:**
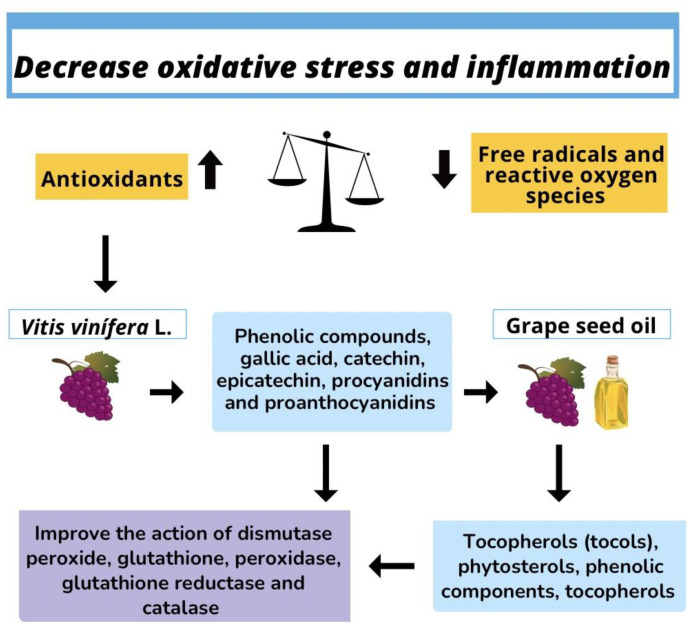
Decrease oxidative stress and inflammation applications of grape (*Vitis vinífera* L.) seed oil.

Oxidative stress is defined in the literature as an imbalance that occurs when there is a higher concentration of oxidants than antioxidants, which causes an interruption in redox signaling, hindering control and causing molecular damage, i.e., it is an adaptative response (Figure 2) [52]. Thus, the increased production of free radicals and reactive oxygen species in the organism is an amplification factor, i.e., an intensifier, for pathological alterations to appear, in addition to causing oxidative stress, intensifying the inflammatory process [53]. The inflammatory process is a natural defense mechanism to remove harmful stimuli from the body, such as pathogens, irritants, and damaged cells, and start the cicatrization process [54]. It can be classified as acute when referring to a beneficial process that helps to immobilize a lesioned region and allows the rest of the immune system to be activated to heal lesions, or it can be classified as chronic, which can become a problem and not a solution for lesions since chronically inflamed tissues normally induce immune cells in the bloodstream to amplify the inflammatory response, which can destroy healthy tissues in an erroneous attempt to start the repair process (Figure 2) [55].

Much effort is made in today’s research to verify the consequences in biology and medicine, besides seeking ways to minimize or avoid the imbalance between oxidants and antioxidants in an attempt to fight oxidative stress and inflammation [54]. Among the endogenous antioxidant compounds that best combat oxidative stress there are dismutase peroxide, glutathione, peroxidase, glutathione reductase, and catalase; among the exogenous antioxidants are vitamin E, carotenoids, polyphenols, and vitamin C [56]. *Vitis vinifera* contains various bioactive compounds that can enhance antioxidant and anti-inflammatory actions, such as high levels of phenolic compounds gallic acid, catechin, epicatechin, procyanidins, and proanthocyanidins (Table 4) [57].

Kapcsándi et al. [58] reported the total polyphenol content in grape seed oil to be between 0.24 and 1.13 mg GAE/g and the total antioxidant content to be between 0.12 and 0.78 mg of the Trolox equivalent antioxidant capacity (TEAC)/g, demonstrating that it is a potent protector against oxidative stress. Mollica et al. [39] assessed the biological activity of the oil and found that it could inhibit the enzymes α-glycosidase, α-amylase, α-tyrosinase and cholinesterase (ChE), demonstrate anti-inflammatory activity by lipoxygenase (5-LOX) and stimulate the liberation of macrophages in trials of lipopolysaccharides (LPS). In addition, they reported a significant polyphenol (199.31 mg GAE/g), antioxidant (1036.98 mg TE/g), and enzymatic inhibitor (α-tyrosinase, 151.30 mg KAE/g) content, providing strong evidence for the potential use of grape seed oil as a functional food supplement in the human diet to mitigate alterations caused by cell stress and inflammation.

Wijekoon et al. [57] noted that grape seed can be an important source of anthocyanins, flavones, flavonols, and stilbenes (resveratrol), highlighting the relevance of consuming grape varieties with seeds as a functional food. Argon et al. [59] confirmed that grape seed oil contains bioactive components such as tocopherols (tocols), phytosterols, phenolic components, and other fat-soluble compounds; tocopherols are powerful natural liposoluble antioxidants, and carotenoids also have antioxidant effects. 

Shaban et al. [60] administered 150 mg of grape seed oil per kg of body weight in mice with carcinoma cells. After 8 days of treatment, they observed the induction of apoptosis and potential redox, which reduced inflammation and oxidative stress. Concerning inflammation, Niknami et al. [43] tested grape seed oil at doses of 2, 4, and 8 mL/kg (via gavage) in male Wistar rats with ulcerative colitis. Administering the oil 2 h before the induction of colitis and continuing for 4 days, they observed significantly reduced colon weight, ulcer index, and total colitis index compared with the control group. The results demonstrate that grape seed oil has a potent anti-inflammatory effect and, thus, may be indicated to help in the treatment or prevention of ulcerative colitis. 

Bocsan et al. [14] aimed to verify whether grape seed oil could have an anti-inflammatory effect on ischemia induced by isoproterenol in rats, and they observed that a 14-day dose of 4 mL/kg/day significantly reduced pro-inflammatory (IL-6 and TNF-α) and anti-inflammatory (IL-10) cytokines.

Millan-Linares et al. [61] investigated the effects of the unsaponifiable fraction of *V. vinifera* seed oil on oxidative and inflammatory responses using the fluorescence-activated cell sorting (FACS) analysis of reverse transcriptase followed by a real-time quantitative reverse-transcription polymerase chain reaction (RT-qPCR) and an enzyme-linked immunoassay (ELISA). They observed that at doses of 10–100 μg mL^−1^ of grape seed oil, there was a deviation in the plasticity of monocytes to the monocytes CD14+ and CD16 + +, non-classic anti-inflammatory monocytes, and the reduced inflammatory competence of human primary monocytes treated with LPS, lowering the gene expression and secretion of TNF-α, IL-1β, and IL-6. Furthermore, the unsaponifiable fraction of grape seed oil showed intense activity in eliminating reactive oxygen species (ROS), significantly lowering the nitrite levels, with considerably diminished gene No. 2 expression. The study of Zhoa et al. [62] also verified trunk cells derived from human adipose stem cells (hASCs) during the administration of 200 μM grape seed oil. On the tenth day, they observed a significant reduction in the pro-inflammatory gene expression induced by LPS in human adipocytes and the secretion of cytokines (IL-6 and IL-8).

## 5. Effects of *Vitis vinifera* L. Seeds on Obesity and Metabolic Alterations

It is known that adipose tissue has several physiological functions and participates in intense metabolic activity, contributing to energy balance and hormone regulation (insulin and catecholamines) in addition to the nutritional state under prolonged fasting or increased energy expenditure (Table 5) [63], mediated by biosynthesis, uptake and the storage of tryglycerides from food, and non-lipidic substrates such as carbohydrates, favoring anabolic action, also called lipogenic activity [64]. In addition, it contributes to the liberation of stored triglycerids, benefiting TG hydrolysis in long-chain fatty acids or glycerol, and can be mobilized to tissues and promote catabolic action, i.e., lipolitic activity [65].

Alterations in the energy homeostasis dynamics, i.e., more lipogenic activity and less lipolytic activity, can lead to abnormal fat buildup, causing hypertrophy and/or hyperplasia of adipocytes, and thus obesity [66]. This triggers alterations to the hormone regulators of metabolism and satiety (insulin and leptin) and increases the expression of pro-inflammatory cytokines such as monocyte chemoattractant protein-1 (MCP-1/CCL-2), tumor necrosis factor-alpha (TNF-α) and interleukin-6 (IL-6) [65,67]. Inflammation is involved in many chronic multisystemic conditions, including obesity, atherosclerosis, and diabetes mellitus type 2; many studies have demonstrated the effects of the consumption of fatty acids, especially polyunsaturated linoleic and ÿ-linolenic acids, and its effects on the treatment of metabolic and pathological alterations caused by obesity (Table 6) [67].

**Figure 3:**
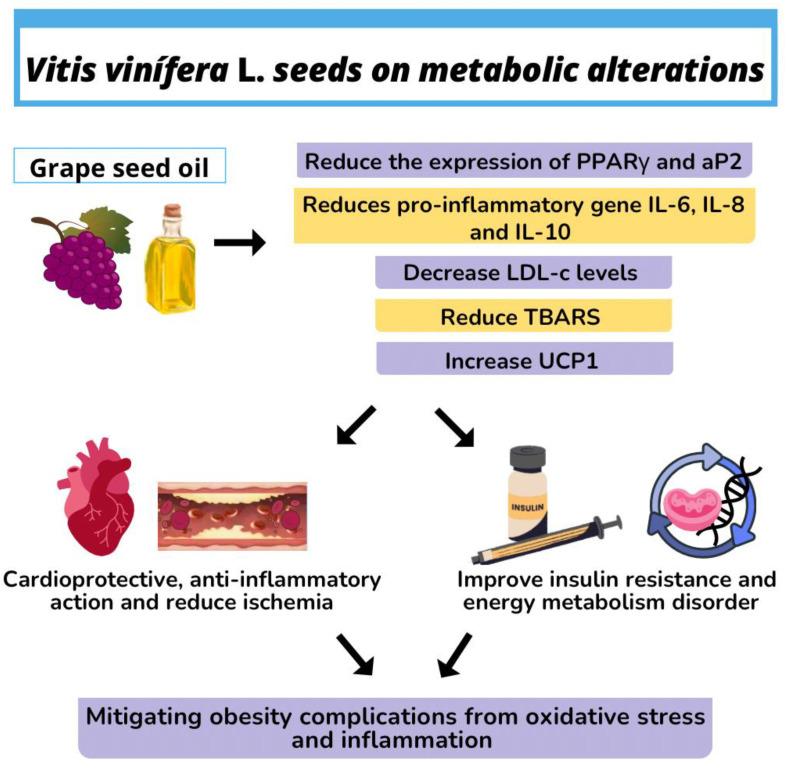
*Vitis vinífera* L. seeds on metabolic alterations applications of grape (*Vitis vinífera* L.) seed oil.

Grape seed oil contains tocotrienols, with significative quantities of α- and γ-tocotrienol (T3), which significantly reduce the expression of mRNA protein, which is crucial for adipogenesis (e.g., PPARγ and aP2); it sharply reduces the pro-inflammatory gene expression (IL-6 and IL-8) [62]. In addition, the organic fatty acids in grape seed oil, such as malic, tartaric, and oxalic acids, help to lower LDL-c levels [13]. This function is further enhanced by the presence of polyphenols such as flavanols, catechin, and epicatechin, which may be correlated with the inhibition of arterial thrombosis, and compounds such as flavones, isoflavones, and anthocyanins, which have anti-inflammatory activity and are involved in the prevention of pathologies associated with weight gain (Figure 3) [68].

Bocsan et al. [13] evaluated the cardioprotective and anti-inflammatory effects of grape seed oil on ischemia induced by isoproterenol (ISO) in rats (4 mg/kg/day grape seed oil by gavage for 14 days). They observed reduced ventricular conduction, preventing alterations in ECG, changes in biological and inflammatory parameters after myocardial heart stroke induced by ISO, and cardioprotective effects in ischemia induced by ISO, indicating that it may be a potential option for the treatment of cardiovascular diseases (Figure 3).

Mohanna et al. [69] demonstrated for the first time in an experimental model that 12 weeks of supplementation with *V. vinifera* seed oil diminished the expression of the M1 marker and total F4/80 macrophages in white adipose tissue, significantly reduced pro-inflammatory adipokines in the serum and mRNA levels of inflammatory adipokines in the white adipose tissue, and increased the gene expression of uncoupling protein 1 (UCP1), which causes increased cell metabolism, thus elevating energy expenditure. These results indicate new beneficial effects and establish the potential use of grape seed oil to prevent obesity and its comorbidities.

Other in vivo studies have shown that Wistar rats with streptozotocin-induced diabetes treated with *V. vinifera* seed oil (25 mg/kg body weight) for 40 days presented a significant decline in serum glucose concentration, and in levels of plasma triglyceride (TG), low-density lipoprotein (LDL-c) and very-low-density lipoprotein (VLDL-c), demonstrating that grape seed oil can improve dyslipidemia and hyperglycemia in diabetic rats [70]. Li et al. [71] assessed the potential of grape seed oil to improve insulin resistance and the energy metabolism disorder in C57BL/6J mice when fed a fat-rich diet and observed that after supplementing with oil at 25.9% weight/total weight for 15 weeks, the animals showed increased energy. An effect on insulin resistance was also observed, which could be associated with the protective effect of hexokinase and α-glycosidase activity and improved leptin resistance, suggesting that polyphenols may be the most critical factor in regulating insulin resistance and demonstrating that grape seed oil has a beneficial influence on the correlation between insulin resistance, energy metabolism, and hyperlipidemia.

Mice fed a hyperlipidic diet supplemented with 15.5 g grape seed oil per 100 g of food for 8 weeks showed lower body weight gain and white adipose tissue weight, as well as reduced plasma levels of glucose and total cholesterol and improved glucose tolerance. In addition, remarkable antioxidant properties were observed, with low plasma levels of TBARS (substances reactive to thiobarbituric acid, biomarkers of lipidic peroxidation), in addition to the reduced production of IL-6 and IL-10. This allows us to conclude that *V. vinifera* seed oil can be considered as a functional oil capable of mitigating obesity complications from oxidative stress and inflammation [72]. Studies on the use of grape seed oil in the treatment of obesity are scarce; however, despite there being little evidence regarding weight reduction, grape seed oil has an effect on blood lipid parameters and reduced blood glycemia in obese mice (Table 5) [73].

**Table 5:** Compounds related to antioxidant and anti-inflammatory effects attributed to *Vitis vinifera* seed oil.

Reference	Effects	Related Compounds	Main Results
[59]	Antioxidant Anti-inflammatory	- Total antioxidant content - Flavonoids - Vitamin E - Vitamin C	- TEAC: 0.14 and 1.16 mg/g - DPPH: 31.0 and 45.3%. - Total phenolic content: 48–360 mg GAE/kg - Phytosterols: β-sitosterol: 83.5–91.9 mg/100 g Stigmasterol: 30.5–32.6 mg/100 g Campesterol: 12.7–13.7 mg/100 g - Vitamin E: 223 mg α (5,7,8-trimetyltocol) β (5,8-dimetyltocol) γ (7,8-dimetyltocol) δ (8-metyltocol) - Carotenoids: 56.7 ppm β-carotene β-cryptoxanthine α-carotene
[58]	Antioxidant	Polyphenols Total antioxidant content	Polyphenols: 0.24–1.13, e.g., GAE/g Total antioxidants: 0.12 and 0.78 mg TEAC/g
[39]	Antioxidant Anti-inflammatory	Polyphenols Total antioxidant content	- Inhibit enzymes α-glycosidase, α-amylase, α-tirosinase and cholenisterase - Stimulate liberation of macrophages - Polyphenols: 199.31 mg GAE/g - Antioxidant content: 1036.98 mg TE/g
[57]	Antioxidant Anti-inflammatory	- Anthocyanins - Flavones - Flavanols - Stilbenes (resveratrol)	- 70% linoleic acid - Total cinnamic acid derivates: 89.2 µg/g - Total hydroxybenzoic acid: 31.9 µg/g - Total flavan-3-ols: 33.6 µg/g - Total flavanols: 85.6 µg/g - Total flavones: 19.7 µg/g - Total stilbenes (resveratrol): 13.9 µg/g - Total anthocyanins: 190.9 µg/g

*Vitis vinifera* seed oil was proven to significantly inhibit the proliferative growth of human colon cancer cells (HT-29) under different treatment times in vitro, and this protection is attributed to polyphenolic compounds, which have the capacity to inhibit kinase proteins, blocking cell proliferative transduction signals (Table 6).

**Table 6:** Summary of the effects of *Vitis vinifera* L seed oil on obesity and metabolic alterations.

Reference	Effects	Object/ Population	Period	Study Design	Main Results
[14]	- Cardioprotective - Antioxidant - Anti-inflammatory	Wistar rats	14 days	Group 1: Saline solution 0.4 mL/100 g Group 2: Saline solution 0.4 mL/100 g Group 3: *Nigella sativa* seed oil 0.4 mL/100 g Group 4: *Vitis vinifera* seed oil 0.4 mL/100 g	↓ ventricular conduction ↓ IL-6, IL-1 β and TNF-α ↓ CK-Mb Prevented cardiotoxic effect of ISO
[43]	- Anti-inflammatory - Antioxidant - Anticarcinogenic	In vitro	8 days	Evaluated inhibitory effect of 150 mg *Vitis vinifera* seed oil on growth of MCF-7 breast cancer cells	↑ apoptosis ↓ inflammation ↓ redox potential (E _h_) ↓ CD44 cells
[61]	- Anti-inflammatory - Antioxidant	Newborn human monocytes	24 h	Newborn human monocytes used to analyze effects of unsaponifiable fraction of *Vitis vinifera* seed oil (10–100 µg/mL) on oxidative and inflammatory responses using FACS, RT-qPCR and ELISA	↓ CD14 ↑ surface expression of CD16 in human primary monocytes treated with LPS ↓ gene expression and secretion of TNF-α, IL-1β and IL-6
[62]	- Anti-obesogenic - Anti-inflammatory	In vitro	12 days	Trunk cells derived from primary human adipose tissue treated with 200 μM *Vitis vinifera* seed oil	↓ expression of mRNA ↓ adipogenic proteins (PPARγ and aP2)
[69]	- Anti-obesogenic - Anti-inflammatory	Swiss mice	12 weeks	Group 1: Diet A04-10 with 3% of energy as soybean oil Group 2: High-fat diet with 21% additional energy in milk cream fat Group 3: Diet + *Vitis vinifera* seed oil Group 4: Diet + *Vitis vinifera* seed oil enriched with 200 mg/kg/day resveratrol	↓ expression of marker M1 ↓ expression of macrophagss F4/80 in white adipose tissue ↓ pro-inflammatory adipokines of soro ↓ levels of mRNA from inflammatory adipokines in white adipose tissue ↑ gene expression of uncoupling protein 1 (UCP1)
[70]	- Glycemic control - Lipid control	Wistar rats	40 days	Group 1: Diabetic rats treated with 25 mg/kg *Vitis vinifera* seed oil Group 2: Diabetic rats treated with saline solution	↓ serum glucose ↓ triglycerides ↓ low-density liproprotein (LDL-c) ↓ very-low-density liproprotein (VLDL-c)
[71]	- Anti-obesogenic - Antioxidant - Anti-inflammatory - Glycemic control	C57BL/6J mice	15 weeks	Group 1: Control group Group 2: Diet rich in lard (25.93%) Group 2: Diet rich in corn oil (25.93%) Group 3: Diet rich in *Vitis vinífera* seed oil (25.93%)	↑ energy rate ↓ insulin resistance ↓ fasting glucose ↓ serum insulin ↓ glucagon concentration ↓ leptin resistance
[72]	- Anti-obesogenic - Antioxidant - Anti-inflammatory	Swiss mice	8 weeks	Group 1: control diet Group 2: High-fat diet (HFD) with 100% of lipidic content as lard Group 3: HFD with 50% of lipidic content as grape seed oil (HG) Group 4: HFD with 50% of lipidic content as SLs containing capric acid produced from grape seed oil (HG-MCT)	↓ body weight gain ↓ adiposity ↓ serum glucose ↓ total cholesterol ↓ plasma TBARS ↓ production of IL-6 and IL-10
[73]	- Anti-inflammatory - Antioxidant	Wistar rats	2 h before colitis induction, after 4 consecutive days	Group 1: Sham (normal) oral physiological serum/without colitis induction Group 2: Induced colitis (negative control), saline solution/colitis induction Groups 3, 4 and 5: Three doses (50, 100 and 200 mg/kg) of *Vitis vinifera* seed extract with colitis induction Groups 6, 7 and 8: Three doses (2, 4 and 8 mL/kg) of *Vitis vinifera* seed oil with colitis induction Groups 9 and 10: Reference (positive control), prednisone (4 mg/kg) or masalamine (100 mg/kg) with colitis induction	↓ colon weight ↓ ulcer index ↓ total colitis index ↓ oxidative stress ↓ inflammation
[74]	- Antioxidant - Antiproliferative	In vitro		Human HT-29 colorectal adenocarcinoma cells, 24 h incubation, using 2 g *Vitis vinifera* seed oil	↓ proliferation of human colon cancer cells (HT-29)
[75]	- Cardioprotective - Anti-inflammatory - Antidiabetic	Wistar rats	24 weeks	Group 1: Control diet Group 2: Experimental model of MetS Group 3: Diet-induced MetS treated with Grape-derived stilbene concentrate (GDSC) (from the 14th to the 24th week) Group 4: induced MS treated with GDSC (from the 19th to the 24th week)	↓ abdominal fat ↓ average glucose level ↓ triglycerides ↑ GLUT4 ↑ PPAR-γ (GDSC treatment from the 14th week of the experiment) ↓ PPAR-γ (GDSC treatment from the 24th week of the experiment) ↓ TLR4 concentration ↓ CRP level
[76]	- Cardioprotective - Antioxidant	Wistar rats	7 days	Group 1: Control diet Group 2: aqueous solution of cobalt chloride (CoCL_2_) Group 3: Aqueous solution of cobalt chloride (CoCL_2_) + 0.25 mL/kg of the extract of grape polyphenols together with 0.5 mL/kg of water Group 4: Aqueous solution of cobalt chloride (CoCL_2_) + 2.5 mL/kg of red wine “health”	↓ free radical oxidation of lipids (TBA-AP) ↑ Catalase (CLA) ↑ Peroxidase (PLA) ↑ Enzyme superoxide dismutase (SOD)
[76]	- Cardioprotective - Antioxidant	rats		Group 1: Control diet Group 2: Metabolic syndrome + 0.5 g/L wine diluted with water Group 3: Metabolic syndrome + 1.0 g/L wine diluted with water Group 4: Metabolic syndrome + 2.5 g/L wine diluted with water	↓ free radical oxidation of lipids (TBA-AP) ↑ Enzyme superoxide dismutase (SOD) ↑ Peroxidase (PLA) ↑ Catalase (CLA)
[76]	- Cardioprotective - Antioxidant	Wistar rats	2 weeks	Group 1: Control diet Group 2: Standard food and drinking water and were undergo bloodletting within the first week of the experiment Group 3: Undergo bloodletting within the first week of the experiment and received sparkling red wine (0.5 mL/100 g of body weight) diluted with water Group 4: Did not undergo bloodletting and received sparkling red wine (0.5 mL/100 g of body weight) diluted with water	↑ free radical oxidation of lipids (TBA-AP) ↑ Enzyme superoxide dismutase (SOD) ↑ Peroxidase (PLA)

↑: increased; ↓: decreased.

## 6. Conclusions

The wine industry generates large quantities of residues, with grape seeds standing out among them since their extracted oil presents antioxidant and anti-inflammatory activities. Seed oil primarily comprises polyunsaturated fatty acids such as linoleic acid, vitamin E, and phytosterols, along with hydrophilic phenols, and shows promise as a nutritional compound and a valuable therapeutical substance, described in the literature as exhibiting antiobesogenic, antidiabetogenic, and anti-cancer activity. 

Nevertheless, studies confirming such therapeutic qualities based on clinical experiments remain scarce. Most results are based on the nutritional and phytochemical composition of *Vitis vinifera* seed oil and not exclusively on the findings from original studies. Thus, more studies are needed to confirm such results and determine the mechanisms of action involved in the indicated therapeutical properties. 

## Figures and Tables

**Table 1:** Nutrients and fiber in *Vitis vinifera* L. seeds (g/100 g; minimum and maximum values) [8,9,24].

Component	Quantity per 100 g
Carbohydrates (g)	18.2–19.8
Proteins (g)	8.7–9.8
Lipids (g)	10.6–16.7
Monounsaturated fatty acids (g)	8.8–22.1
Polyunsaturated fatty acids (g)	67.2–78.2
Saturated fatty acids (g)	7.0–12.8
Fiber (g)	40.2–43.7
Energy (Kcal/100 g)	216.8–237.4

**Table 2:** Proportions of fatty acids in *Vitis vinifera* L. seed oil (%) [8,9,14].

Fatty Acids	Percentage (%)
C8:0 (Caprilic acid)	0.01
C12:0 (Lauric acid)	0.01
C14:0 (Myristic acid)	0.05
C15:0 (Pentadecilic acid)	0.01
C16:0 (Palmitic acid)	6.7
C17:0 (Heptadecanoic acid)	0.06
C18:0 (Estearic acid)	3.8
C20:0 (Arachidic acid)	0.16
C16:1 (n-7) (Palmitoleic acid)	0.2
C18:1 *cis* (n-9) (Oleic acid)	14.8
C20:1(n-9) (Gadoleic acid)	0.40
C18:2 *cis* (n-6) (Linolenic acid)	74.2
C18:3 (n-3) (α-Linolenic acid)	0.11
Saturated fatty acids (SFAs)	10.6
Monounsaturated fatty acids (MUFAs)	14.9
Polyunsaturated fatty acids (PUFAs)	74.3
n-3 PUFAs (ω-3)	0.2
n-6 PUFAs (ω-6)	74.7

**Table 3:** Macro- and microelements of *Vitis vinifera* L. seed oil (mg per 1000 g seeds; minimum and maximum values) [16,27,28,29,30].

Component	mg/1000 g
Potassium	4347.8–9492.6
Iron	29.9–73.8
Phosphorus	2277.6–3232.4
Calcium	1249.1–2073.9
Magnesium	249.1–2073.9
Zinc	8.2–15.9
Manganese	2.0–11.5
Sulphur	8.6–15.2

**Table 4:** Main phytochemical compounds in *Vitis vinifera* L. seed oil [10,11,13,14].

Components	Quantity per 100 g
Flavonoids (mg) [10]	59.7
Epicatechin (mg) [10]	130.4
Catechins (mg) [11]	414.0
Procyanidins (mg) [11]	2.5
Phenolics (µg) [13]	73.0
Gallic acid (µg) [10]	77.0
Condensed tannins (mg CE) [14]	14.0
Vitamin E	
α-Tocopherols (mg)	86–244
β-Tocopherols (mg)	38–48
γ-Tocopherols (mg)	17–29
α-Tocotrienols (mg)	216–319
β-Tocotrienols (mg)	4–18
γ-Tocotrienols (mg)	499–1575
Vitamin C	46.0–179.2
Vitamin A	
Carotenoids (mg)	27.0–48.0
β-carotene (ppm)	33.9–59.8

## Data Availability

Not applicable.
